# Moderate-vigorous physical activity attenuates premature senescence of immune cells in sedentary adults with obesity: a pilot randomized controlled trial

**DOI:** 10.18632/aging.204458

**Published:** 2022-12-29

**Authors:** Xiang-Ke Chen, Chen Zheng, Stephen Heung-Sang Wong, Alvin Chun-Hang Ma

**Affiliations:** 1 Department of Health Technology and Informatics, The Hong Kong Polytechnic University, Hung Hom, Hong Kong SAR, China; 2 Department of Sports Science and Physical Education, The Chinese University of Hong Kong, Sha Tin, Hong Kong SAR, China

**Keywords:** physical inactivity, obesity, immune aging, premature senescence, senolytic

## Abstract

Despite the well-known senolytic effects of physical exercise on immune cells in older adults, the effect of physical activity (PA) on premature immune senescence in sedentary adults with obesity remains largely unknown. This pilot study aimed to investigate the role of objectively measured physical behaviors and Fitbit watch-based free-living PA intervention in premature senescence of immune cells in sedentary adults with obesity. Forty-five participants were recruited in the cross-sectional analysis, and forty of them further participated in the randomized controlled trial. We found that objectively measured moderate–vigorous PA was independently and inversely correlated with the expression of p16^INK4a^ and p21^Cip1^ in the peripheral blood mononuclear cell (PBMCs) of adults with obesity; however, chronological age, body mass index, body fat, maximal oxygen consumption, light PA, sedentary behaviors, and sleep duration were not. More importantly, the 12-week PA intervention mitigated the elevated p16^INK4a^ levels in PBMCs, though it showed no effect on p21^Cip1^ and senescence-associated secretory phenotypes. Taken together, physical inactivity is an independent determinant of premature senescence in immune cells, while the 12-week PA intervention is a promising strategy to alleviate premature immune senescence in adults with obesity.

## INTRODUCTION

A sedentary or inactive lifestyle is one of the leading causes of mortality, accelerated aging, and a wide array of age-related diseases, such as metabolic diseases, cardiovascular diseases, and cancer. In contrast, being physically active has long been recognized as a safe and effective “medicine” for the aging population [1]. Nevertheless, according to the World Health Organization (WHO), approximately one in five men and one in three women globally fail to meet the recommendations for weekly minimal moderate–vigorous physical activity (MVPA) [2]. The world is aging rapidly, with around 13% of the world population (one billion) aged 60 years and above, as reported by WHO in 2020 [3]. This has led to a dramatic increase in the burden of aging and aging-related diseases. Many studies have revealed that physical inactivity is a critical determinant of life and health spans [4, 5]. Additionally, due to the effectiveness and cost-effectiveness of physical activity (PA), being physically active during aging, known as “active aging,” is a promising strategy to reduce the burden of aging and age-related diseases [6, 7]. In this context, a sedentary or inactive lifestyle serves as a therapeutic target for accelerated aging and multiple age-related diseases; however, its adverse effects on the health of the aging population and the relevant underlying mechanisms are not yet fully understood.

Cellular senescence, an irreversible state of cell cycle arrest that occurs under conditions of stress, is a key mechanism underlying aging. The aberrant accumulation of senescent cells that express atypical levels of p16^INK4a^ and p21^Cip1^, vital senescent markers, contributes to premature aging and age-related diseases [8]. Thus, senescent cells are therapeutic targets for cellular senescence in aging and age-related diseases and premature senescence in diseases that occur at a younger age, such as obesity [9]. Notably, a recent study revealed that senescent immune cells could trigger systemic and premature aging of organs and tissues throughout the body; in contrast, alleviation of immune senescence slows down whole-body aging [10]. Therefore, the removal or attenuation of senescent immune cells, also known as senolytics or senostatics, is a potential therapy for premature aging and age-related diseases at not only the cellular but also the whole-organism level. Senolytics are a novel class of medicine that targets cellular senescence in various conditions, which have shown great potential in treating many diseases, such as diabetic kidney disease and idiopathic pulmonary fibrosis [11–13]. However, no senolytic targeting immune senescence is currently available, though many senolytic candidates have been discovered and are currently being tested in clinical trials [12, 13].

Previous studies have shown that self-reported exercise frequency is negatively correlated with p16^INK4a^ levels in T lymphocytes, independent of age [14, 15]. This implies that premature senescence of immune cells may account for sedentary lifestyle-related accelerated aging and age-related diseases. However, studies on objectively measured physical behaviors, such as PA, standing, sedentary behaviors, and sleep, have not yet been reported. Nonetheless, our systematic review and other studies have uncovered the senolytic effect of physical exercise or activity interventions on the immune system in older individuals, and potentially in individuals with obesity [16, 17]. Undoubtedly, physical exercise is an effective senolytic against aging and age-related diseases in older adults. However, the senolytic effects of habitual PA and PA interventions on premature senescence of immune cells, particularly in sedentary adults with obesity, remain unknown. Compared with administering treatments against severe diseases at a later stage, alleviating accelerated aging at a younger age is a better strategy for prolonging the health span and reducing the risk of diseases in the aging population. Therefore, this pilot study aimed to investigate the role of objectively measured physical behaviors and PA intervention in premature senescence of immune cells in sedentary adults with obesity. The findings generated by this study may contribute to understanding the associations between PA and premature senescence and provide a novel approach against accelerated aging in the aging population.

## RESULTS

**Figure 1:**
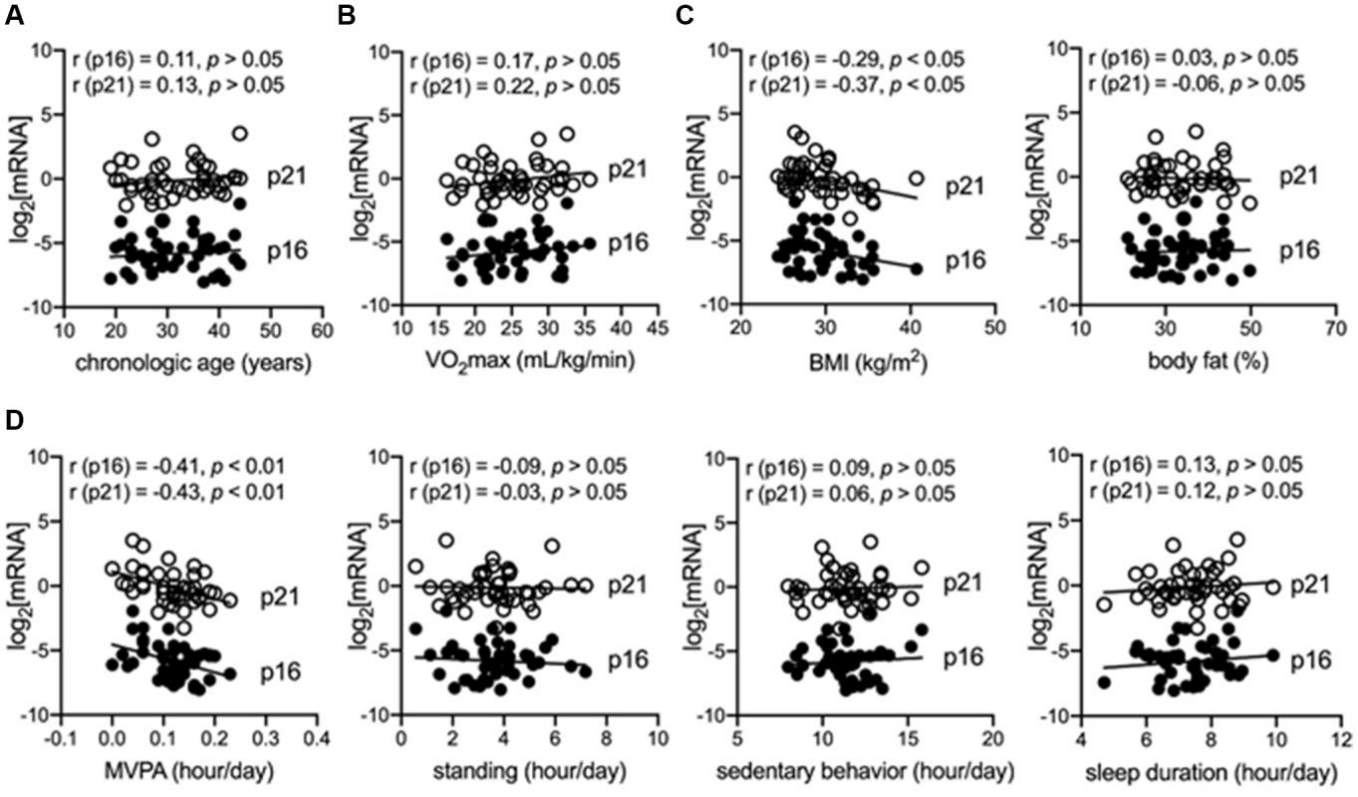
**MVPA is independently and negatively correlated with senescent markers in immune cells of adults with obesity.** (**A**) Correlation between chronological age and senescent markers (log_2_-transformed p16^INK4a^ and p21^Cip1^) in peripheral blood mononuclear cells (PBMCs). (**B**) Correlation between maximum oxygen consumption (VO_2_max) and senescent markers in PBMCs. (**C**) Correlation between body mass index (BMI) or body fat and senescent markers in PBMCs. (**D**) Correlation between objectively measured physical behaviors, including moderate–vigorous physical activity (MVPA), standing, sedentary behaviors, sleep duration, and senescent markers in PBMCs.

The demographic, anthropometric, and behavioral characteristics of the included 45 participants (age, 31.71 ± 7.30 years) are shown in Table 1. The cross-sectional analysis indicated that chronological age, maximal oxygen consumption (VO_2_max), body fat, and blood pressures (diastolic blood pressure and systolic blood pressure) were not associated with the log_2_-transformed p16^INK4a^ and p21^Cip1^ levels in peripheral blood mononuclear cell (PBMCs) in sedentary adults with obesity (*p* > 0.05) (Figure 1A–1C, Tables 2, 3; Supplementary Figure 1). Notably, MVPA was independently and reversely correlated with log_2_-transformed p16^INK4a^ (B = −8.83, 95% CI = −15.98 to −1.68, *p* = 0.02) and p21^Cip1^ (B = −8.30, 95% CI = −14.74 to −1.85, *p* = 0.01) levels in PBMCs; however, no significant correlation was found in the other physical behaviors, including light PA (LPA), vigorous PA, standing, sedentary behaviors, and sleep duration (*p* > 0.05) (Figure 1D and Table 2; Supplementary Figure 1). Although the body mass index (BMI) and daily steps were also significantly correlated with log_2_-transformed p16^INK4a^ and p21^Cip1^ levels in PBMCs (*p* < 0.05) (Figure 1C; Supplementary Figure 1), the correlation became non-significant after adjusting for other factors (*p* > 0.05) (Table 2). These findings suggested that insufficient MVPA is a major driver of cellular senescence of immune cells in sedentary adults with obesity.

**Table 1:** Characteristics of participants.

**Characteristics**	**Mean ± SD**
Number (female, %)	45 (42%)
Age, years	31.71 ± 7.30
Body mass index, kg/m^2^	29.54 ± 3.56
Body weight, kg	84.45 ± 14.19
Body fat, %	33.87 ± 7.45
Waist circumference, cm	96.87 ± 15.35
Diastolic blood pressure, mmHg	80.89 ± 9.20
Systolic blood pressure, mmHg	113.56 ± 13.46
VO_2_max, ml/kg/min	25.27 ± 4.82
Sedentary time, hour/day	11.47 ± 1.74
Light physical activity, min/day	48.97 ± 19.53
Moderate to vigorous physical activity, min/day	7.07 ± 3.23
Vigorous physical activity, min/day	1.13 ± 1.80
Standing, hour/day	3.61 ± 1.38
Steps, steps/day	7975 ± 2876

Abbreviation: VO_2_max: maximal oxygen consumption.

**Table 2:** Determinants of premature senescence in sedentary adults.

**Measurement**	**Log(p16^INK4a^)**	***P* value**	**Log(p21^Cip1^)**	***P* value**
	**B (95% CI)**		**B (95% CI)**	
**Demographics**				
Age, years	0.05 (−0.01 to 0.10)	.12^a^	0.05 (−0.003 to 0.10)	.06^a^
**Anthropometry**				
BMI, kg/m^2^	−0.08 (−0.18 to 0.03)	.17^a^	−0.10 (−0.19 to 1.41)	.05^a^
Body weight, kg	−0.01 (−0.04 to 0.01)	.32^b^	−0.02 (−0.04 to 0.01)	.17^b^
Body fat, %	0.02 (−0.04 to 0.07)	.57^b^	−0.004 (−0.05 to 0.04)	.87^b^
VO_2_max, ml/kg/min	0.03 (−0.04 to 0.11)	.41^a^	0.04 (−0.03 to 0.11)	.24^a^
**Behavior**				
SB, hr/day	0.16 (−0.09 to 0.42)	.21^a^	0.15 (−0.08 to 0.38)	.21^a^
LPA, hr/day	−0.26 (−1.91 to 1.39)	.76^c^	−0.37 (−1.87 to 1.12)	.63^c^
MVPA, hr/day	−8.83 (−15.98 to −1.68)	.02^a^	−8.30 (−14.74 to −1.85)	.01^a^
Steps, 1000 steps/day	−0.14 (−0.29 to 0.01)	.06^c^	−0.10 (−0.24 to 0.04)	.15^c^
Sleep duration, hr/day	0.20 (−0.19 to 0.58)	.32^a^	0.13 (−0.23 to 0.48)	.49^a^

Abbreviations: BMI: body mass index; VO_2_max: maximal oxygen consumption; SB: sedentary behaviors; LAP: low-intensity physical activity; MVPA: moderate-vigorous intensity physical activity. ^a^Generalized Linear Models Analysis, Model 1: age, MVPA, BMI, SB, sleep duration, VO_2_max. ^b^Generalized Linear Models Analysis, Model 2: BMI in model 1 was replaced by indicated measurement. ^c^Generalized Linear Models Analysis, Model 3: MVPA in model 1 was replaced by indicated measurement.

**Table 3:** Anthropometric, behavioral, and senescent responses to physical activity intervention.

**Measurement**	**Mean (SD)**				***P* value^a^**		
	**Pre-control**	**Post-control**	**Pre-PA**	**Post-PA**	**Interaction effect**	**Group effect**	**Time effect**
**Anthropometry**							
BMI, kg/m^2^	28.82 (2.75)	28.91 (3.14)	29.69 (3.82)	29.25 (4.34)	.69	.95	.80
Body weight, kg	81.53 (14.01)	81.87 (15.56)	85.50 (12.08)	84.11 (12.79)	.59	.74	.74
Body fat, %	33.00 (5.54)	33.45 (5.73)	33.97 (9.00)	32.65 (9.60)	.52	.61	.75
VO_2_max, ml/kg/min	25.56 (4.34)	26.61 (6.60)	25.91 (5.40)	29.27 (7.74)	.28	.26	.04
**Behavior**							
SB, hr/day	11.32 (1.31)	11.12 (2.32)	10.93 (2.17)	10.95 (2.5)	.44	.66	.38
LPA, hr/day	0.86 (0.31)	0.94 (0.51)	0.73 (0.39)	0.77 (0.31)	.53	.42	.78
MVPA, hr/day	0.13 (0.04)	0.11 (0.08)	0.10 (0.08)	0.20 (0.07)	.003	.004	.03
Steps, steps/day	8490 (2557)	8430 (4133)	7082 (3812)	11544 (3988)	.04	.04	.01
Sleep duration, hr/day	7.86 (1.02)	7.35 (0.91)	7.48 (0.74)	7.40 (0.77)	.56	.68	.07
**Cellular senescence**							
-Log(p16^INK4a^)	6.10 (1.36)	−3.76 (1.97)	5.55 (1.40)	−2.86 (1.50)	.04	.07	<.001
-Log(p21^Cip1^)	0.43 (1.54)	2.67 (1.30)	−0.18 (1.32)	3.11 (1.58)	.14	.99	<.001
-Log(IL-1β)	−7.21 (1.12)	−10.58 (0.85)	−7.50 (0.87)	−11.07 (0.92)	.77	.52	.06
-Log(IL-6)	−10.12 (1.26)	−7.56 (1.24)	−10.19 (1.23)	−7.97 (1.27)	.26	.08	<.001
-Log(TNF-α)	−6.89 (0.81)	−6.83 (0.83)	−6.75 (0.61)	−6.66 (0.69)	.90	.69	.58
IL-1β, pg/ml	19.16 (5.86)	19.20 (4.42)	23.94 (8.74)	23.28 (8.95)	.18	.91	.19
IL-6, pg/ml	16.45 (2.79)	16.72 (4.36)	18.13 (3.06)	16.27 (2.21)	.09	.23	.21
IL-8, pg/ml	21.33 (6.56)	20.51 (4.80)	22.74 (7.26)	19.28 (3.15)	.12	.05	.01
CCL2, pg/ml	107.61 (36.98)	95.64 (25.56)	134.30 (68.46)	97.32 (26.47)	.13	.46	<.001
ICAM-I, ng/ml	1224.30 (503.12)	1262.33 (408.97)	1099.13 (404.18)	1117.45 (419.60)	.76	.53	.38
VEGF, pg/ml	23.04 (7.89)	22.30 (10.75)	24.06 (12.65)	19.53 (7.17)	.34	.19	.003
PAI-I, ng/ml	172.54 (82.01)	206.03 (36.23)	211.02 (90.27)	235.24 (57.12)	.77	.18	.07

Abbreviations: PA: physical activity intervention; BMI: body mass index; VO_2_max: maximal oxygen consumption; SB: sedentary behaviors; LAP: low-intensity physical activity; MVPA: moderate-vigorous intensity physical activity; IL: interleukin; TNF: tumor necrosis factor; CCL2: C–C motif chemokine ligand 2; ICAM-I: intercellular adhesion molecule 1; VEGF: vascular endothelial growth factor; PAI-I: plasminogen activator inhibitor-1. ^a^Generalized Estimated Equation Analysis.

Furthermore, a randomized controlled trial (RCT) was conducted to investigate the 12-week PA intervention on the anthropometric, behavioral, and senescent markers in adults with obesity. Significant increases in MVPA (group effect, *p* = 0.004) and steps (group effect, *p* = 0.04 [activPAL™]; group effect, *p* < 0.01 [Fitbit Watch]) were observed in the PA intervention group compared with those in the control group (Table 3; Supplementary Figures 2, 3). In contrast, the other physical behaviors, including sedentary behaviors, LPA, and sleep duration, remained unchanged after the 12-week PA intervention (*p* > 0.05) (Table 3). However, the change in habitual MVPA showed no effect on the anthropometric measures, including BMI, body weight, body fat, and VO_2_max (*p* > 0.05). More importantly, the 12-week PA intervention significantly attenuated the elevated log_2_-transformed p16^INK4a^ levels in PBMCs (interaction effect, *p* = 0.04) (Table 3; Supplementary Figure 4A). However, the intervention had no impact on the expression of p21^Cip1^, interleukin (IL)-1β, IL-6, and tumor necrosis factor (TNF)-α in PBMCs and serum IL-1β, IL-6, IL-8, C-C motif chemokine ligand 2 (CCL2), intercellular adhesion molecule 1 (ICAM-I), vascular endothelial growth factor (VEGF), and plasminogen activator inhibitor-1 (PAI-I) (*p* > 0.05) levels, which are also known as senescence-associated secretory phenotypes (SASPs) (Table 3; Supplementary Figure 4B). Intriguingly, while MVPA was inversely correlated with both p16^INK4a^ and p21^Cip1^ levels in PBMCs, the 12-week PA intervention showed a sole effect on p16^INK4a^ but not on p21^Cip1^ and other SASPs. No adverse event was reported during the 12-week PA intervention.

## DISCUSSION

Premature senescence accelerates aging and elevates the risk of many diseases in the aging population, including cancer, metabolic diseases, and neurological diseases, in an age-independent manner [18, 19]. Thus, effective senolytics or senostatics against premature senescence will contribute to a lower burden of aging and age-related diseases worldwide. Although chronological age has long been identified as the main driver of senescence, the determinants of premature senescence in adults with obesity are less studied [20]. This pilot cross-sectional analysis and RCT demonstrated that a sedentary lifestyle, especially the lack of MVPA, was a major contributing factor to premature senescence of immune cells in adults with obesity in age-, VO_2_max-, and BMI-independent manners. Furthermore, the 12-week PA intervention significantly attenuated the elevated p16^INK4a^ levels in the immune cells of sedentary adults with obesity. These findings highlight the importance of an active lifestyle in maintaining a youthful immune system and the senolytic or senostatic effects of PA on premature senescent immune cells in sedentary adults with obesity.

A strong correlation between log_2_-transformed p16^INK4a^ mRNA level in immune cells and chronological age was previously reported in an aging population [14]. However, our findings suggested that chronological age and cardiorespiratory fitness (VO_2_max) were not the major drivers of cellular senescence of immune cells in sedentary adults with obesity. Instead, physical inactivity, especially lacking MVPA, was a determinant of premature senescence of immune cells in sedentary adults with obesity. However, sedentary behaviors, LPA, standing, and sleep duration were not determinants of premature senescence. Physical inactivity is a potential mechanism underlying the adverse effects of a sedentary lifestyle on healthy aging via accelerating immune system senescence [10]. Expectedly, chronological age was not a driver of premature senescence in a relatively younger population. Premature senescence is commonly triggered by other stressful conditions, such as an unhealthy lifestyle [21]. Besides, cardiorespiratory fitness (VO_2_max) has long been associated with a higher proportion of senescent immune cells, which are distinguished by the surface markers of immune cells in middle-aged but not in young adults [22]. While the surface markers of immune cells can also classify senescent cells by the stage of the cell cycle, such as the CD28^-^CD57^+^KLRG1^+^ terminally-differentiated memory T cell subset, more reliable markers involved in the pathway of cellular senescence are needed [23]. By using golden standard markers of cellular senescence, including p16^INK4a^ and p21^Cip1^, our findings showed that VO_2_max is not a determinant of cellular senescence in the immune cells of adults with obesity. Previous cohort studies have reported that self-reported exercise frequency is independently and negatively correlated with p16^INK4a^ levels in immune cells. However, the evidence is too preliminary since only a simple question about exercise was used in the questionnaire [14, 15]. To the best of our knowledge, this is the first study to report that insufficient objectively measured MVPA is a major factor contributing to the cellular senescence of immune cells in sedentary adults with obesity. Compared with previous studies, we identified MVPA, a specific type of PA, as a key determinant of cellular senescence in immune cells. We used activPAL™ to objectively measure physical behaviors, which provided a more precise therapeutic target against immune aging for the aging population, with more solid evidence than that of previous studies [14, 15]. In addition, sedentary behavior is another vital physical behavior-based risk factor for unhealthy aging, independent of PA [24]. Surprisingly, LPA and sedentary behaviors were not major contributing factors to the premature senescence of immune cells in sedentary adults with obesity. Collectively, inadequate MVPA was a vital driver of premature senescence of immune cells in sedentary adults with obesity; in contrast, chronological age, cardiorespiratory fitness, LPA, and sedentary behavior showed subtle effects.

Our previous systematic review and meta-analysis confirmed that chronic physical exercise is a senolytic for cellular senescence in immune cells [16]. Moreover, a novel study also reported that a 12-week constructed exercise training program effectively reduced senescent markers of immune cells and SASPs in older adults [17]. However, the effect of free-living PA on cellular senescence of immune cells in the aging population, especially premature senescence in adults with obesity, remains largely unknown. The current study was the first to demonstrate the senolytic effects of PA intervention on senescent immune cells in sedentary adults with obesity. Unlike the previous interventional studies using structured exercise programs [16, 17], the current study provides evidence that increasing daily MVPA is an effective strategy against premature senescence of immune cells in sedentary adults with obesity. More importantly, the habitual PA of participants was improved by our intervention program, which will bring more prolonged benefits than previous physical exercise programs. Alleviation of cellular senescence in immune cells is potentially a key molecular mechanism underlying “active aging,” and the “exercise as medicine” [7] approach can go a long way in alleviating premature immune senescence in adults with obesity. Additionally, this study focused on the premature senescence of immune cells in sedentary adults with obesity, who are neglected in currently available senolytic studies. Premature senescence may account for accelerated senescence and the elevated burden of aging and age-related diseases. A previous interventional study reported that exercise lowered elevated p16^INK4a^ and p21^Cip1^ levels in immune cells and circulating SASPs in older individuals [17]. However, elevated p16^INK4a^ and declined p21^Cip1^ levels were observed in sedentary adults with obesity, which probably indicated early-stage accumulation of senescence immune cells during aging. This is because p16^INK4a^ maintains the senescent phenotypes while p21^Cip1^ initiates cellular senescence [25]. Moreover, consistent with the findings of previous animal studies, our results demonstrated that PA intervention only exerted a senolytic effect on p16^INK4a^ in the immune cells but not on p21^Cip1^ and SASPs of sedentary adults with obesity [26, 27]. This suggested distinct senescent phenotypes and senolytic effects of exercise between older adults and adults with obesity. Specifically, the senolytic effects of PA in older adults involved both the inhibition of the production of senescent cells (p21^Cip1^) and the removal of excess senescent cells (p16^INK4a^), whereas it only reduced excess senescent cells in sedentary adults with obesity. It should be noted that cellular senescence is not only a key molecular mechanism behind aging but also a vital biological process that is essential for tissue repair, cancer suppression, and health maintenance [28]. Our results show that PA intervention is a relatively safe senolytic for sedentary adults with obesity during aging. This senolytic only targets p16^INK4a^+ excess senescent cells without affecting the p21^Cip1^-regulated initiation of cellular senescence [25].

This study has several limitations. The main limitation is that this small-scale pilot study only included 45 participants with a narrow age range, which limits our ability to investigate the diversity of key senescent determinants in various age groups. In addition, the levels of p16^INK4a^ and p21^Cip1^ in PBMCs measured in this study remained at the mRNA level. While it is a conventional and reliable method in the determination of cellular senescence in immune cells [14, 15], findings based on the level of p16^INK4a^ and p21^Cip1^ at the protein level are more convincing. In addition, our primary protocol deviations from the planned trial are as follows: we included senescent markers as outcomes for studying immune senescence in sedentary adults with obesity and removed the PA + middle-to-high-intensity exercise group due to the coronavirus disease 2019 pandemic. Nevertheless, our study provides a feasible anti-aging strategy involving increasing daily steps, which will contribute to healthy aging and a reduced burden of aging and age-related diseases for individuals and society, respectively. Nevertheless, large-scale RCTs on the senolytic effect of PA intervention targeting premature senescence in different populations at various age stages, such as healthy sedentary adults, middle-aged adults, and adults with type-2 diabetes, are still needed. These studies may contribute to developing exercise prescriptions for promoting healthy aging and reducing the burden of age-related diseases in the accelerated aging populations worldwide.

## CONCLUSION

Physical inactivity at a younger age is an independent determinant of premature senescence in immune cells, potentially leading to accelerated aging of the whole body. A 12-week free-living PA intervention targeting MVPA is a senolytic for premature immune senescence in sedentary adults with obesity. Being physically active by increasing daily steps is an effective and cost-effective strategy to slow the aging process and reduce the burden of aging and age-related diseases.

## MATERIALS AND METHODS

### Participants

**Figure 2:**
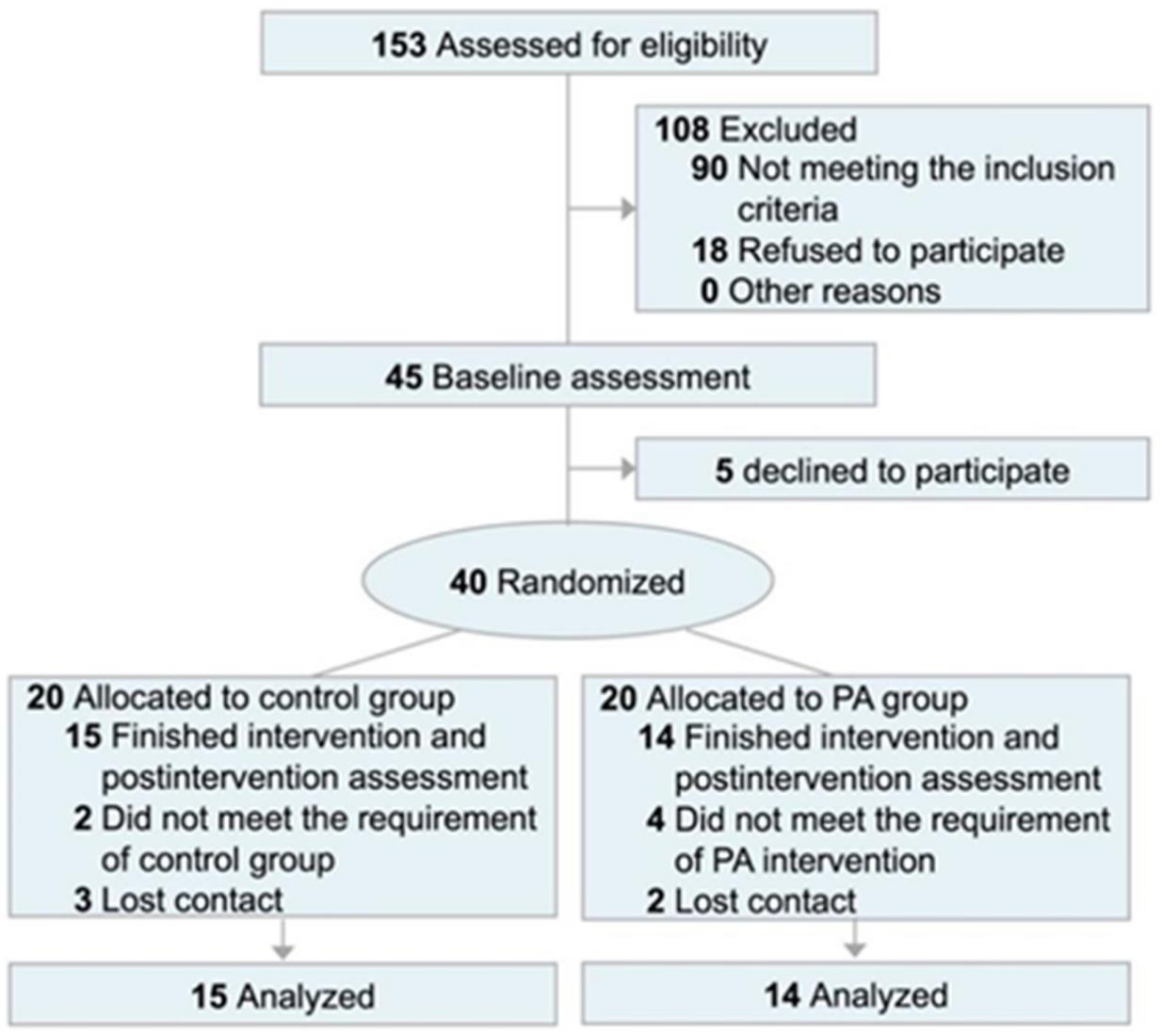
**CONSORT flow diagram.** Abbreviation: PA: physical activity intervention.

Between September 2020 and December 2020, 153 adults were contacted and screened telephonically. Subsequently, 63 adults were invited and screened through laboratory visits. Finally, 45 participants who met the inclusion criteria were included in a cross-sectional baseline assessment (Figure 2). Inclusion criteria for adults with a sedentary lifestyle were as follows: 1) Chinese adults; 2) aged between 18 and 45 years; 3) physically inactive (less than 150-minute MVPA per week) and with prolonged sedentary behavior (sitting time over eight hours per day) as screened by the Chinese version of the International Physical Activity Questionnaire-Short Version [29] and activPAL™ (PAL Technologies, Glasgow, UK); 4) BMI ≥ 25 kg/m^2^; 5) blood pressure less than 140/90 mmHg, 6) non-smoker and non-drinker of alcohol; 7) without cardiovascular diseases, such as heart diseases, vascular diseases, or diabetes; 8) no medical history of physical injuries in the last three months; and 9) not taking any medicine in the last three months. In addition, Gpower software (GPower Software Inc., University of Kiel, Kiel, Germany) was used to calculate the sample size based on the data derived from a previous RCT in the elderly [17] and a medium effect size (Cohen’s f) of p16^INK4a^ = 0.283 was determined. Consequently, approximately fourteen participants in each group were estimated to achieve the effect size (0.283) by using a two-arm pretest-posttest design at a two-sided significance level of 5% and at a power of 80% (*F* tests, ANOVA: repeated measures, within-between interaction). Therefore, 28 participants in total were required for a two-arm RCT, and finally, more than 40 participants were recruited, as approximately 10 of the invited participants were estimated to be lost to follow-up. Of the 45 participants, 40 participated in the two-arm RCT (20 in each group) of a 12-week Fitbit watch-based PA intervention, while five declined to participate (Figure 2). All the experimental protocols were reviewed and approved by the Joint Chinese University of Hong Kong-New Territories East Cluster Clinical Research Ethics Committee (CREC Ref. No.:2020.551-T). Written informed consent was obtained from each participant. This RCT was registered at the Chinese Clinical Trial Registry (ChiCTR2000039033).

### Anthropocentric measurements

The anthropocentric measurements of participants, including height, weight, BMI calculation, and body fat, were performed as described previously [30]. For the VO_2_max test, a modified Bruce protocol was employed for the participants using the treadmill according to a previous study [31].

### Physical behaviors measured by activPAL™

Habitual physical behaviors, including physical activities, sedentary behaviors, standing, sleep duration, and daily steps, were measured using activPAL™, in which the small accelerator was placed in the front mid-line of the right thigh of participants for 24 h for five days consecutively days with at least one day of the weekend in pre- and post- 12-week PA intervention (Supplementary Figure 2A). The data was analyzed using PALanalysis v 8.0 (PAL Technologies, Glasgow, UK) as previously described (Supplementary Figure 2A–2C) [30].

### Fitbit watch-based PA intervention

The participants in the PA intervention group underwent a 12-week PA intervention using the Fitbit Inspire 2 watch (Fitbit, San Francisco, USA) (Supplementary Figure 3A) [32], while participants in the control group were asked to maintain their original lifestyle. The intervention for physical activities/steps was conducted in a free-living setting, which was monitored via the Web-based activities tracking system provided by Fitbit (Supplementary Figure 3A–3D). The goal for the PA was set at over 12,000 steps per day for at least 5 days per week. The researchers messaged participants who had not yet achieved the goal daily.

### Blood sampling and PBMCs isolation

Venous blood samples were collected from an antecubital vein in the right arm at pre- and post-intervention by a nurse between 8:00 and 10:00 am. Participants were asked to fast for eight hours before the blood collection and avoid alcohol, caffeine, and exercise for more than 24 h. The serum was separated from the blood sample by using serum tubes (BD Company, New Jersey, USA) through centrifugation. Furthermore, PBMCs were isolated from blood samples with EDTA using Ficoll-Paque PLUS (GE Healthcare, Uppsala, Sweden) through gradient centrifugation at 2000 rpm for 30 min at room temperature. Serum and isolated PBMCs were then stored at −80°C for further analysis.

### Quantitative PCR

Total RNA was first extracted from the PBMCs using RNAiso Plus (Takara Bio Inc, Shiga, Japan) and reverse transcribed to cDNA using cDNA Reverse Transcription Kit (Takara Bio Inc, Shiga, Japan) according to the manufacturer’s protocol. The qPCR was finally conducted in the Applied Biosystems QuantStudio 7 Flex Real-Time PCR System using the SYBR green reagents (Takara Bio Inc, Shiga, Japan) and primers listed in Supplementary Table 1 according to the manufacturer’s protocol.

### Multiplex assay and ELISA

The senescence-associated secretory phenotypes (IL-1β, IL-6, IL-8, CCL2, ICAM-I, VEGF, and PAI-I) in the serum were measured using the Luminex multiplex assay (R&D Systems Minneapolis, MN, USA) or ELISA (ImmunoDiagnostics Limited, Hong Kong, China), as previously described [30]. For the Luminex multiplex assay, a Bio-Plex 200 System™ (Bio-Rad Laboratories, Hercules, CA, USA) was used to read the flow-based magnetic beads after incubation with serum samples.

### Statistical analyses

Data in this study are presented as mean ± standard deviation (S.D.). Pearson correlation, generalized linear model analysis, and generalized estimated equation analysis were utilized where appropriate using SPSS version 26.0 (IBM Corp., Armonk, N.Y., USA), as previously described [30]. The log_2_-transformed mRNA level was used in this study as previously described [14]. A two-tailed *p*-value < 0.05 was considered statistically significant.

## Supplementary Materials

Supplementary Figures

Supplementary Table 1

## References

[1] Pedersen BK, Saltin B. Exercise as medicine - evidence for prescribing exercise as therapy in 26 different chronic diseases. Scand J Med Sci Sports. 2015 (Suppl 3); 25:1–72. 10.1111/sms.12581 26606383

[2] Guthold R, Stevens GA, Riley LM, Bull FC. Worldwide trends in insufficient physical activity from 2001 to 2016: a pooled analysis of 358 population-based surveys with 1·9 million participants. Lancet Glob Health. 2018; 6:e1077–86. 10.1016/S2214-109X(18)30357-7 30193830

[3] World Health Organization (WHO). Decade of healthy ageing: baseline report. 2020.

[4] Ekelund U, Steene-Johannessen J, Brown WJ, Fagerland MW, Owen N, Powell KE, Bauman A, Lee IM, and Lancet Physical Activity Series 2 Executive Committe, and Lancet Sedentary Behaviour Working Group. Does physical activity attenuate, or even eliminate, the detrimental association of sitting time with mortality? A harmonised meta-analysis of data from more than 1 million men and women. Lancet. 2016; 388:1302–10. 10.1016/S0140-6736(16)30370-1 27475271

[5] Lear SA, Hu W, Rangarajan S, Gasevic D, Leong D, Iqbal R, Casanova A, Swaminathan S, Anjana RM, Kumar R, Rosengren A, Wei L, Yang W, et al. The effect of physical activity on mortality and cardiovascular disease in 130 000 people from 17 high-income, middle-income, and low-income countries: the PURE study. Lancet. 2017; 390:2643–54. 10.1016/S0140-6736(17)31634-3 28943267

[6] Lee IM, Shiroma EJ, Lobelo F, Puska P, Blair SN, Katzmarzyk PT, and Lancet Physical Activity Series Working Group. Effect of physical inactivity on major non-communicable diseases worldwide: an analysis of burden of disease and life expectancy. Lancet. 2012; 380:219–29. 10.1016/S0140-6736(12)61031-9 22818936 PMC3645500

[7] Bauman A, Merom D, Bull FC, Buchner DM, Fiatarone Singh MA. Updating the Evidence for Physical Activity: Summative Reviews of the Epidemiological Evidence, Prevalence, and Interventions to Promote "Active Aging". Gerontologist. 2016 (Suppl 2); 56:S268–80. 10.1093/geront/gnw031 26994266

[8] Ben-Porath I, Weinberg RA. The signals and pathways activating cellular senescence. Int J Biochem Cell Biol. 2005; 37:961–76. 10.1016/j.biocel.2004.10.013 15743671

[9] van Deursen JM. The role of senescent cells in ageing. Nature. 2014; 509:439–46. 10.1038/nature13193 24848057 PMC4214092

[10] Yousefzadeh MJ, Flores RR, Zhu Y, Schmiechen ZC, Brooks RW, Trussoni CE, Cui Y, Angelini L, Lee KA, McGowan SJ, Burrack AL, Wang D, Dong Q, et al. An aged immune system drives senescence and ageing of solid organs. Nature. 2021; 594:100–5. 10.1038/s41586-021-03547-7 33981041 PMC8684299

[11] Kirkland JL, Tchkonia T, Zhu Y, Niedernhofer LJ, Robbins PD. The Clinical Potential of Senolytic Drugs. J Am Geriatr Soc. 2017; 65:2297–301. 10.1111/jgs.14969 28869295 PMC5641223

[12] Hickson LJ, Langhi Prata LGP, Bobart SA, Evans TK, Giorgadze N, Hashmi SK, Herrmann SM, Jensen MD, Jia Q, Jordan KL, Kellogg TA, Khosla S, Koerber DM, et al. Senolytics decrease senescent cells in humans: Preliminary report from a clinical trial of Dasatinib plus Quercetin in individuals with diabetic kidney disease. EBioMedicine. 2019; 47:446–56. 10.1016/j.ebiom.2019.08.069 31542391 PMC6796530

[13] Justice JN, Nambiar AM, Tchkonia T, LeBrasseur NK, Pascual R, Hashmi SK, Prata L, Masternak MM, Kritchevsky SB, Musi N, Kirkland JL. Senolytics in idiopathic pulmonary fibrosis: Results from a first-in-human, open-label, pilot study. EBioMedicine. 2019; 40:554–63. 10.1016/j.ebiom.2018.12.052 30616998 PMC6412088

[14] Liu Y, Sanoff HK, Cho H, Burd CE, Torrice C, Ibrahim JG, Thomas NE, Sharpless NE. Expression of p16(INK4a) in peripheral blood T-cells is a biomarker of human aging. Aging Cell. 2009; 8:439–48. 10.1111/j.1474-9726.2009.00489.x 19485966 PMC2752333

[15] Song Z, von Figura G, Liu Y, Kraus JM, Torrice C, Dillon P, Rudolph-Watabe M, Ju Z, Kestler HA, Sanoff H, Lenhard Rudolph K. Lifestyle impacts on the aging-associated expression of biomarkers of DNA damage and telomere dysfunction in human blood. Aging Cell. 2010; 9:607–15. 10.1111/j.1474-9726.2010.00583.x 20560902 PMC2910221

[16] Chen XK, Yi ZN, Wong GT, Hasan KMM, Kwan JS, Ma AC, Chang RC. Is exercise a senolytic medicine? A systematic review. Aging Cell. 2021; 20:e13294. 10.1111/acel.13294 33378138 PMC7811843

[17] Englund DA, Sakamoto AE, Fritsche CM, Heeren AA, Zhang X, Kotajarvi BR, Lecy DR, Yousefzadeh MJ, Schafer MJ, White TA, Atkinson EJ, LeBrasseur NK. Exercise reduces circulating biomarkers of cellular senescence in humans. Aging Cell. 2021; 20:e13415. 10.1111/acel.13415 34101960 PMC8282238

[18] Mathon NF, Lloyd AC. Cell senescence and cancer. Nat Rev Cancer. 2001; 1:203–13. 10.1038/35106045 11902575

[19] Palmer AK, Tchkonia T, LeBrasseur NK, Chini EN, Xu M, Kirkland JL. Cellular Senescence in Type 2 Diabetes: A Therapeutic Opportunity. Diabetes. 2015; 64:2289–98. 10.2337/db14-1820 26106186 PMC4477358

[20] Childs BG, Durik M, Baker DJ, van Deursen JM. Cellular senescence in aging and age-related disease: from mechanisms to therapy. Nat Med. 2015; 21:1424–35. 10.1038/nm.4000 26646499 PMC4748967

[21] Ben-Porath I, Weinberg RA. When cells get stressed: an integrative view of cellular senescence. J Clin Invest. 2004; 113:8–13. 10.1172/JCI20663 14702100 PMC300889

[22] Spielmann G, McFarlin BK, O'Connor DP, Smith PJ, Pircher H, Simpson RJ. Aerobic fitness is associated with lower proportions of senescent blood T-cells in man. Brain Behav Immun. 2011; 25:1521–9. 10.1016/j.bbi.2011.07.226 21784146

[23] Koch S, Larbi A, Ozcelik D, Solana R, Gouttefangeas C, Attig S, Wikby A, Strindhall J, Franceschi C, Pawelec G. Cytomegalovirus infection: a driving force in human T cell immunosenescence. Ann N Y Acad Sci. 2007; 1114:23–35. 10.1196/annals.1396.043 17986574

[24] Dogra S, Stathokostas L. Sedentary behavior and physical activity are independent predictors of successful aging in middle-aged and older adults. J Aging Res. 2012; 2012:190654. 10.1155/2012/190654 22997579 PMC3446656

[25] Stein GH, Drullinger LF, Soulard A, Dulić V. Differential roles for cyclin-dependent kinase inhibitors p21 and p16 in the mechanisms of senescence and differentiation in human fibroblasts. Mol Cell Biol. 1999; 19:2109–17. 10.1128/MCB.19.3.2109 10022898 PMC84004

[26] Schafer MJ, White TA, Evans G, Tonne JM, Verzosa GC, Stout MB, Mazula DL, Palmer AK, Baker DJ, Jensen MD, Torbenson MS, Miller JD, Ikeda Y, et al. Exercise Prevents Diet-Induced Cellular Senescence in Adipose Tissue. Diabetes. 2016; 65:1606–15. 10.2337/db15-0291 26983960 PMC4878429

[27] Yoon KJ, Zhang D, Kim SJ, Lee MC, Moon HY. Exercise-induced AMPK activation is involved in delay of skeletal muscle senescence. Biochem Biophys Res Commun. 2019; 512:604–10. 10.1016/j.bbrc.2019.03.086 30910357

[28] He S, Sharpless NE. Senescence in Health and Disease. Cell. 2017; 169:1000–11. 10.1016/j.cell.2017.05.015 28575665 PMC5643029

[29] Macfarlane DJ, Lee CC, Ho EY, Chan KL, Chan DT. Reliability and validity of the Chinese version of IPAQ (short, last 7 days). J Sci Med Sport. 2007; 10:45–51. 10.1016/j.jsams.2006.05.003 16807105

[30] Zheng C, Tian XY, Sun FH, Huang WY, Sheridan S, Wu Y, Wong SH. Associations of Sedentary Patterns with Cardiometabolic Biomarkers in Physically Active Young Males. Med Sci Sports Exerc. 2021; 53:838–44. 10.1249/MSS.0000000000002528 33017350 PMC7969161

[31] McInnis KJ, Balady GJ, Weiner DA, Ryan TJ. Comparison of ischemic and physiologic responses during exercise tests in men using the standard and modified Bruce protocols. Am J Cardiol. 1992; 69:84–9. 10.1016/0002-9149(92)90680-w 1729872

[32] Cadmus-Bertram LA, Marcus BH, Patterson RE, Parker BA, Morey BL. Randomized Trial of a Fitbit-Based Physical Activity Intervention for Women. Am J Prev Med. 2015; 49:414–8. 10.1016/j.amepre.2015.01.020 26071863 PMC4993151

