# Supplementary material for: Moderate-vigorous physical activity attenuates premature senescence of immune cells in sedentary adults with obesity: a pilot randomized controlled trial
Source: Aging (Albany NY). 2022 Dec 29;14(24):10137–52. doi: 10.18632/aging.204458 (PMC9831733; doi:10.18632/aging.204458)
Supplement: Supplementary Figures [file aging-14-204458-s001.pdf]

SUPPLEMENTARY FIGURES

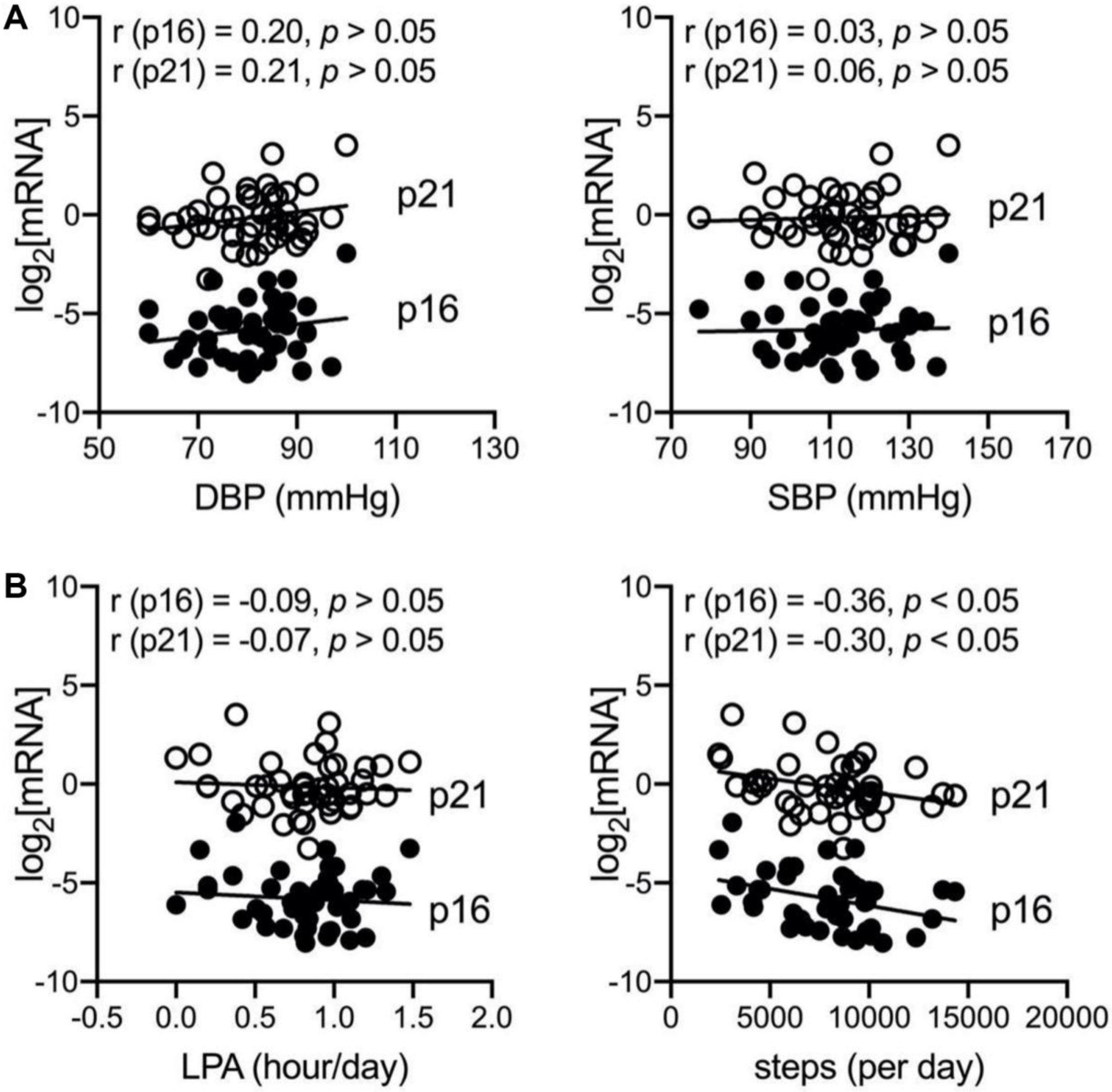

Supplementary Figure 1. Correlation of senescent markers in PBMCs with blood pressure and physical behaviors. (**A**) Blood pressures. (**B**) Physical behaviors. Abbreviations: DBP: diastolic blood pressure; SBP: systolic blood pressure; LPA: light physical activity.

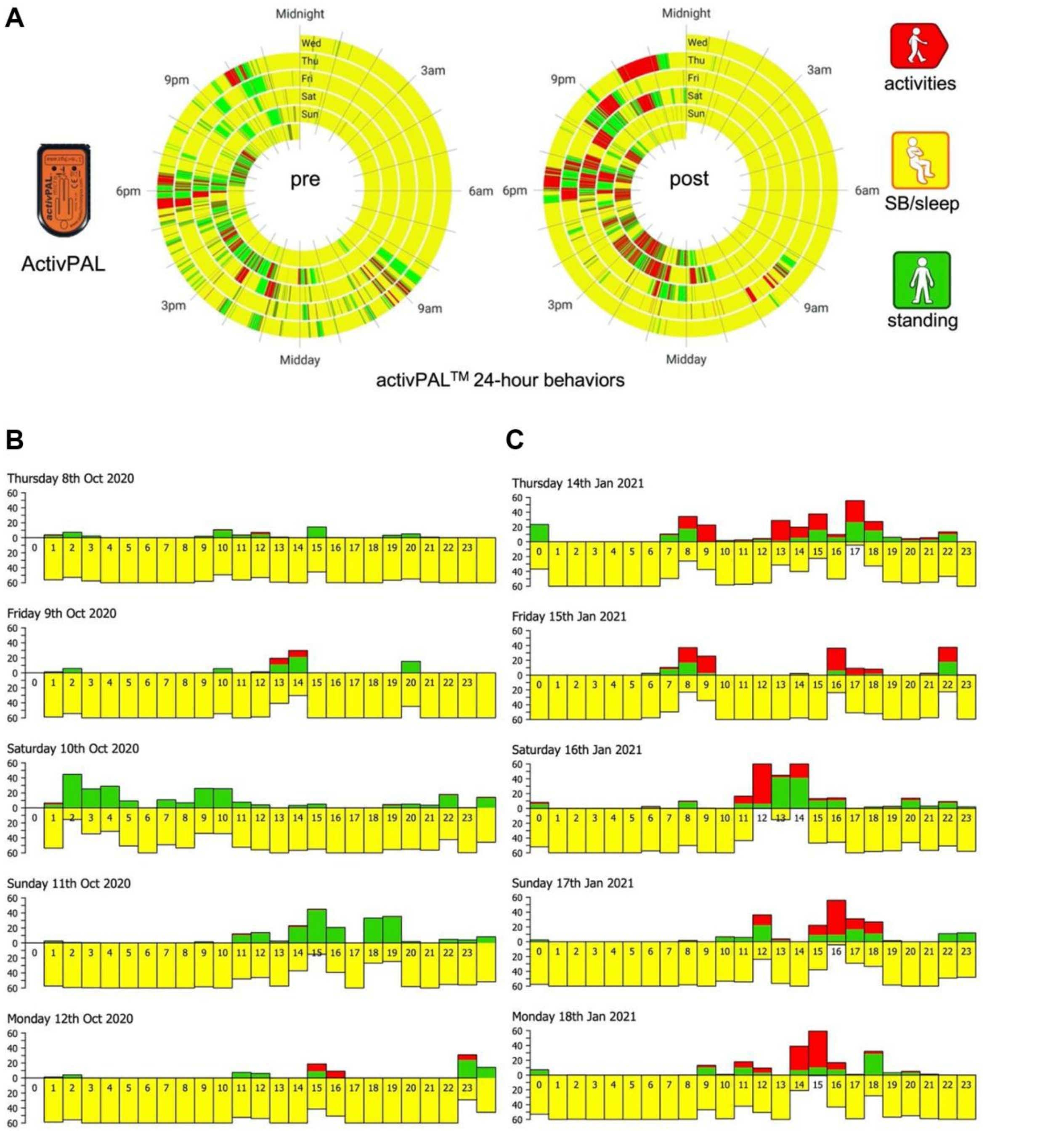

Supplementary Figure 2. Physical behavioral responses measured by activPAL™. (**A**) Individual 24-hour behaviors recorded by activPAL™. (**B, C**) Individual weekly behaviors recorded by activPAL™ before (**B**) and after physical activity (PA) intervention (**C**). Abbreviation: SB: sedentary behaviors.

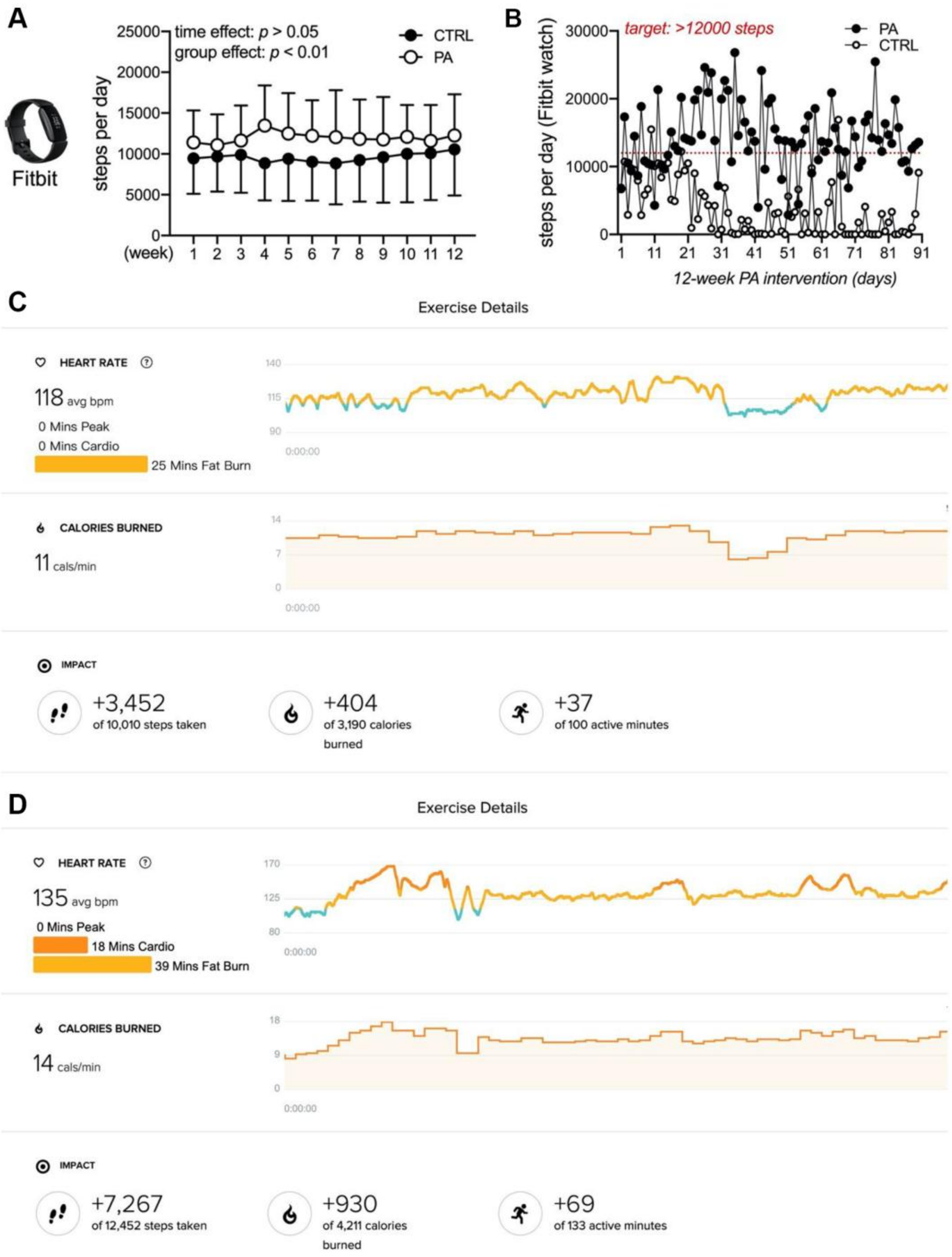

Supplementary Figure 3. Physical activity intervention monitored by Fitbit watch. (**A**) The difference in steps between control (CTRL) and physical activity (PA) intervention groups. (**B**) Individual steps recorded during 12-week PA intervention. (**C**) Individual daily activities recorded by Fitbit watch before PA intervention. (**D**) Individual daily activities recorded by Fitbit watch after PA intervention.

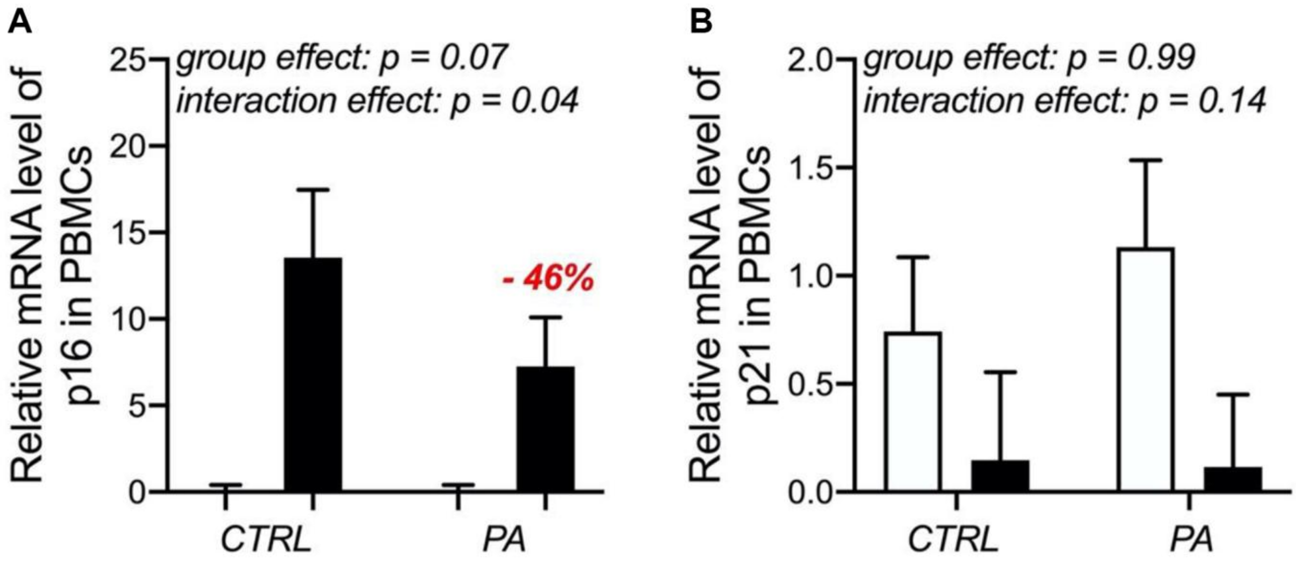

Supplementary Figure 4. The effect of physical activity intervention on the mRNA level of p16^{INK4A} and p21^{Cip1} in PBMCs. (**A**) mRNA level of p16 in peripheral blood mononuclear cells (PBMCs) of control (CTRL) and physical activity (PA) intervention groups before and after 12-week PA intervention. (**B**) mRNA level of p21 in PBMCs of control (CTRL) and physical activity (PA) intervention groups before and after 12-week PA intervention. While bar: pre-intervention; Black bar: post-intervention.
