# Supplementary material for: Moderate-vigorous physical activity attenuates premature senescence of immune cells in sedentary adults with obesity: a pilot randomized controlled trial
Source: Aging (Albany NY). 2022 Dec 29;14(24):10137–52. doi: 10.18632/aging.204458 (PMC9831733; doi:10.18632/aging.204458)
Supplement: Supplementary Table 1 [file aging-14-204458-s002.pdf]

SUPPLEMENTARY TABLE

Supplementary Table 1. Primers used in the present study.

Primers	5′ to 3′
p16^{INK4a}_Fwd	GGGGGCACCAGAGGCAGT
p16^{INK4a}_Rev	GGTTGTGGCGGGGGCAGTT
p21^{Cip1}_Fwd	CCGCCCCCTCCTCTAGCTGT
p21^{Cip1}_Rev	CCCCCATCATATACCCCTAACACA
TNF-α_Fwd	CCTGCCCCAATCCCTTTATT
TNF-α_Rev	CCCTAAGCCCCCAATTCTCT
IL-1β_Fwd	TCCAGGGACAGGATATGGAG
IL-1β_Rev	TCTTTCAACACGCAGGACAG
IL-6_Fwd	AATAACCACCCCTGACCCAAC
IL-6_Rev	AATCTGAGGTGCCCATGCTAC
GAPDH_Fwd	TCTTCTTTTGCGTCGCCAG
GAPDH_Rev	AGCCCCAGCCTTCTCCA

Abbreviations: IL-1β: interleukin-1β; IL-6: interleukin-6; TNF-α: tumor necrosis factor-α; GAPDH: glyceraldehyde 3-phosphate dehydrogenase.
